# Supplementary material for: Transcriptomic Responses to Chilling Reveal Potential Chilling Tolerance Mechanisms in Cucumber
Source: Int J Mol Sci. 2022 Oct 25;23(21):12834. doi: 10.3390/ijms232112834 (PMC9657649; doi:10.3390/ijms232112834)
Supplement: Supplementary file 1 [file ijms-23-12834-s001.zip › ijms-1927225-table S5.pdf]

Table S5 Sequence alignment of variable region in the promoter of *CsCBF1* in 10 cucumber lines

Lines	Sequence
CC	GATAGTAAAAGAGAAAAGTAGAATTTTCGATGGTGAAGAACAAGTCAAAGTGACACATGTCAGTTCCTGA
R1461	GATAGTAAAAGAGAAAAGTAGAATCTTCGATGGTGAAGAACAAGTCAAAGTGACACATGTCAGTTCCTGA
9930	GATAGTAAAAGAGAAAAGTAGAATCTTCGATGGTGAAGAACAAGTCAAAGTGACACATGTCAGTTCCTGA
HN	GATAGTAAAAGAGAAAAGTAGAATCTTCGATGGTGAAGAACAAGTCAAAGTGACACATGTCAGTTCCTGA
XSBN	GATAGTAAAAGAGAAAAGTAGAATCTTCGATGGTGAAGAACAAGTCAAAGTGACACATGTCAGTTCCTGA
HNYH	GATAGTAAAAGAGAAAAGTAGAATCTTCGATGGTGAAGAACAAGTCAAAGTGACACATGTCAGTTCCTGA
HNHP	GATAGTAAAAGAGAAAAGTAGAATCTTCGATGGTGAAGAACAAGTCAAAGTGACACATGTCAGTTCCTGA
ZN	GATAGTAAAAGAGAAAAGTAGAATCTTCGATGGTGAAGAACAAGTCAAAGTGACACATGTCAGTTCCTGA
NT	GATAGTAAAAGAGAAAAGTAGAATCTTCGATGGTGAAGAACAAGTCAAAGTGACACATGTCAGTTCCTGA
HNND	GATAGTAAAAGAGAAAAGTAGAATCTTCGATGGTGAAGAACAAGTCAAAGTGACACATGTCAGTTCCTGA
CC	AGTTGACTCATTGTTTATCAATTTTCATTTTTCAAATATCTTTTTAGTATTTTATGAATTTAAAAAAAAA
R1461	AGTTGACTCATTGTCTATCAATTTTCATTTTTCAAATATCTTTTTAGTATTTTATGAATTTAAAAAAAAA
9930	AGTTGACTCATTGTCTATCAATTTTCATTTTTCAAATATCTTTTTAGTATTTTATGAATTTAAAAAAAAA
HN	AGTTGACTCATTGTCTATCAATTTTCATTTTTCAAATATCTTTTTAGTATTTTATGAATTTAAAAAAAAA
XSBN	AGTTGACTCATTGTCTATCAATTTTCATTTTTCAAATATCTTTTTAGTATTTTATGAATTTAAAAAAAAA
HNYH	AGTTGACTCATTGTCTATCAATTTTCATTTTTCAAATATCTTTTTAGTATTTTATGAATTTAAAAAAAAA
HNHP	AGTTGACTCATTGTCTATCAATTTTCATTTTTCAAATATCTTTTTAGTATTTTATGAATTTAAAAAAAAA
ZN	AGTTGACTCATTGTCTATCAATTTTCATTTTTCAAATATCTTTTTAGTATTTTATGAATTTAAAAAAAAA
NT	AGTTGACTCATTGTCTATCAATTTTCATTTTTCAAATATCTTTTTAGTATTTTATGAATTTAAAAAAAAA
HNND	AGTTGACTCATTGTCTATCAATTTTCATTTTTCAAATATCTTTTTAGTATTTTATGAATTTAAAAAAAAA
CC	AATAGAAAAGGGCCAAAGAGAAAAAAATAAAAAAATAAAAAAAAAGGGAAGGTGTCGTTGATGGCGTGGG
R1461	AATAGAAAAGGGCCAAAGAGAAAAAAATAAAAAAATAAAAAAAAAGGGAAGGTGTCGTTGATGGCGTGGG
9930	AATAGAAAAGGGCCAAAGAGAAAAAAATAAAAAAATAAAAAAAAAGGGAAGGTGTCGTTGATGGCGTGGG
HN	AATAGAAAAGGGCCAAAGAGAAAAAAATAAAAAAATAAAAAAAAAGGGAAGGTGTCGTTGATGGCGTGGG
XSBN	AATAGAAAAGGGCCAAAGAGAAAAAAATAAAAAAATAAAAAAAAAGGGAAGGTGTCGTTGATGGCGTGGG
HNHY	AATAGAAAAGGGCCAAAGAGAAAAAAATAAAAAAATAAAAAAAAAGGGAAGGTGTCGTTGATGGCGTGGG
HNHP	AATAGAAAAGGGCCAAAGAGAAAAAAATAAAAAAATAAAAAAAAAGGGAAGGTGTCGTTGATGGCGTGGG
ZN	AATAGAAAAGGGCCAAAGAGAAAAAAATAAAAAAATAAAAAAAAAGGGAAGGTGTCGTTGATGGCGTGGG
NT	AATAGAAAAGGGCCAAAGAGAAAAAAATAAAAAAATAAAAAAAAAGGGAAGGTGTCGTTGATGGCGTGGG
HNND	AATAGAAAAGGGCCAAAGAGAAAAAAATAAAAAAATAAAAAAAAAGGGAAGGTGTCGTTGATGGCGTGGG
CC	CAGCCAACACAGGCTGGCTCCTTATCATTTAACTCCGTGTTTCAGTGTTTCACTCTGTTGCTTTCAATT-
R1461	CAGCCAACACAGGCTGGCTCCTTATCATTTAACTCCGTGTTTCAGTGTTTCACTCTGTTGCTTTCAATTT
9930	CAGCCAACACAGGCTGGCTCCTTATCATTTAACTCCGTGTTTCAGTGTTTCACTCTGTTGCTTTCAATTT
HN	CAGCCAACACAGGCTGGCTCCTTATCATTTAACTCCGTGTTTCAGTGTTTCACTCTGTTGCTTTCAATTT
XSBN	CAGCCAACACAGGCTGGCTCCTTATCATTTAACTCCGTGTTTCAGTGTTTCACTCTGTTGCTTTCAATTT
HNHY	CAGCCAACACAGGCTGGCTCCTTATCATTTAACTCCGTGTTTCAGTGTTTCACTCTGTTGCTTTCAATTT
HNHP	CAGCCAACACAGGCTGGCTCCTTATCATTTAACTCCGTGTTTCAGTGTTTCACTCTGTTGCTTTCAATTT
ZN	CAGCCAACACAGGCTGGCTCCTTATCATTTAACTCCGTGTTTCAGTGTTTCACTCTGTTGCTTTCAATTT
NT	CAGCCAACACAGGCTGGCTCCTTATCATTTAACTCCGTGTTTCAGTGTTTCACTCTGTTGCTTTCAATTT
HNND	CAGCCAACACAGGCTGGCTCCTTATCATTTAACTCCGTGTTTCAGTGTTTCACTCTGTTGCTTTCAATTT
