# Supplementary material for: Transcriptomic Responses to Chilling Reveal Potential Chilling Tolerance Mechanisms in Cucumber
Source: Int J Mol Sci. 2022 Oct 25;23(21):12834. doi: 10.3390/ijms232112834 (PMC9657649; doi:10.3390/ijms232112834)
Supplement: Supplementary file 1 [file ijms-23-12834-s001.zip › Supplementary_Figures.pdf]

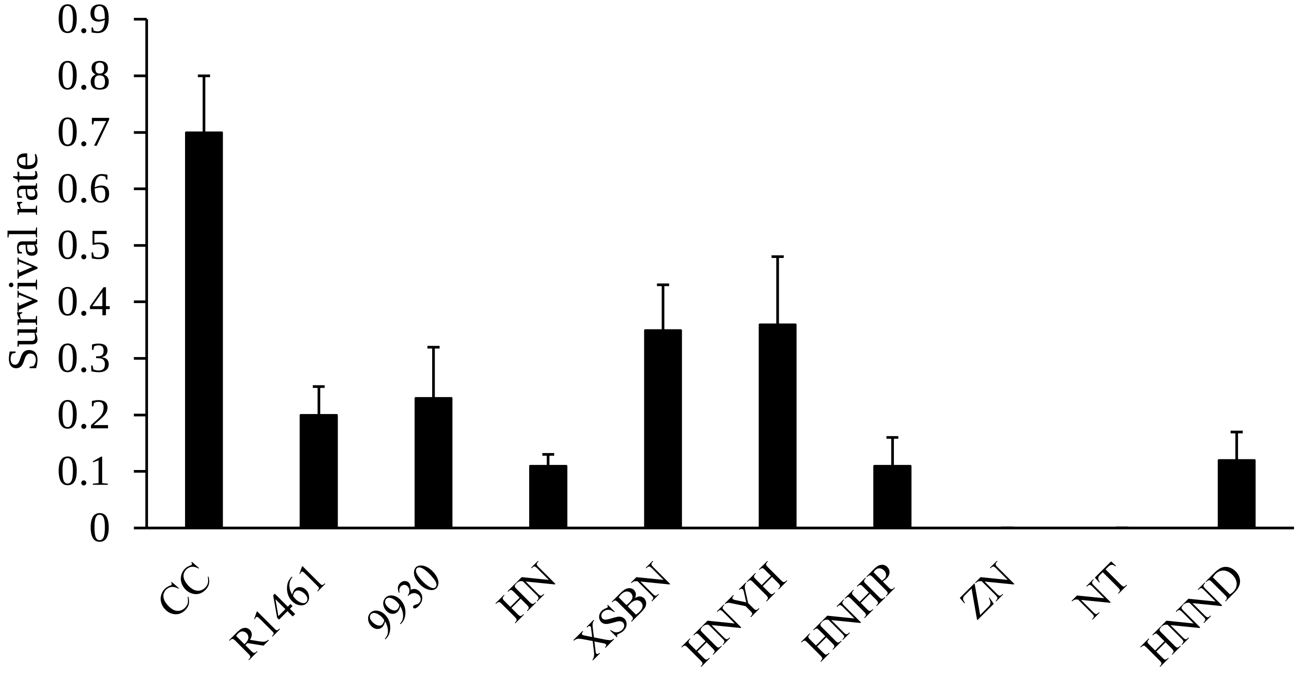

Supplementary Figure S1. Survival rate of Cucumber seedlings after chilling treatment and recovery.

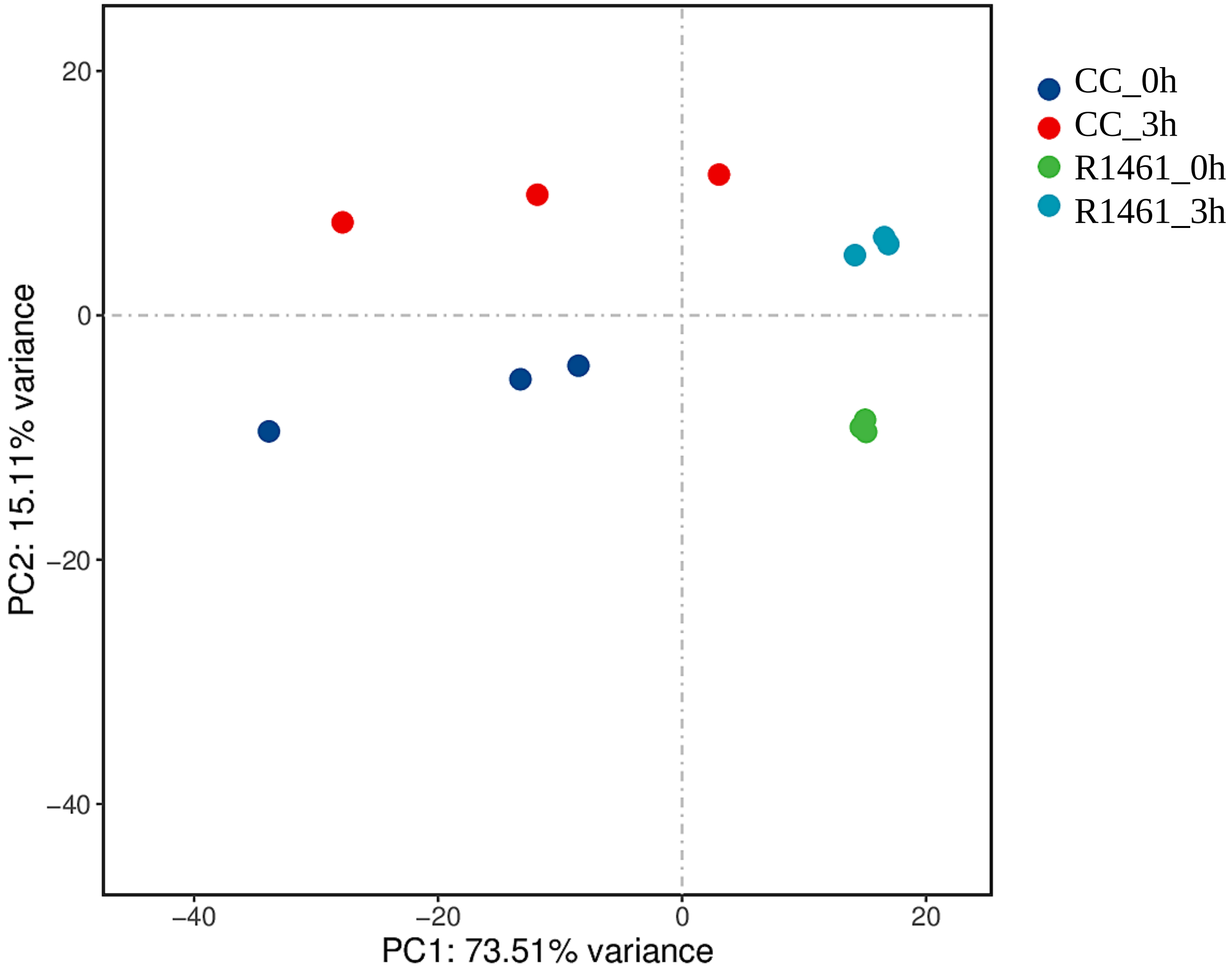

Supplementary Figure S2. Principal component analysis (PCA) of the transcriptomes of CC and R1461 before and after chilling treatment. Seedlings of CC and R1461 were treat at 6ºC for 0h and 3h. RNA-seq were performed on a total of 12 samples (two genotypes, 2 time points and 3 biological replicates). Shown is PC1 and PC2 from PCA analysis on gene expression in these 12 samples.
